# Metformin to Augment Strength Training Effective Response in Seniors (MASTERS): study protocol for a randomized controlled trial

**DOI:** 10.1186/s13063-017-1932-5

**Published:** 2017-04-26

**Authors:** Doug E. Long, Bailey D. Peck, Jenny L. Martz, S. Craig Tuggle, Heather M. Bush, Gerald McGwin, Philip A. Kern, Marcas M. Bamman, Charlotte A. Peterson

**Affiliations:** 1 College of Health Sciences and Center for Muscle Biology, University of Kentucky, Lexington, KY USA; 2 Center for Exercise Medicine and Department of Cell, Developmental, and Integrative Biology, University of Alabama at Birmingham, Birmingham, AL USA; 3 Department of Biostatistics, College of Public Health, University of Kentucky, Lexington, KY USA; 4 Department of Internal Medicine, Division of Endocrinology, and Barnstable Brown Diabetes and Obesity Center, University of Kentucky, Lexington, KY USA

**Keywords:** Metformin, Placebo, Sarcopenia, Aging, Skeletal muscle, Resistance exercise, Inflammation, Medication

## Abstract

**Background:**

Muscle mass and strength are strong determinants of a person’s quality of life and functional independence with advancing age. While resistance training is the most effective intervention to combat age-associated muscle atrophy (sarcopenia), the ability of older adults to increase muscle mass and strength in response to training is blunted and highly variable. Thus, finding novel ways to complement resistance training to improve muscle response and ultimately quality of life among older individuals is critical. The purpose of this study is to determine whether a commonly prescribed medication called metformin can be repurposed to improve the response to resistance exercise training by altering the muscle tissue inflammatory environment.

**Methods/design:**

Individuals aged 65 and older are participating in a two-site, randomized, double-blind, placebo-controlled trial testing the effects of metformin or placebo on muscle size, strength, and physical function when combined with a progressive resistance training program. Participants consume 1700 mg of metformin per day or placebo for 2 weeks before engaging in a 14-week progressive resistance training regimen, with continued metformin or placebo. Participants are then monitored post-training to determine if the group taking metformin derived greater overall benefit from training in terms of muscle mass and strength gains than those on placebo. Muscle biopsies are taken from the vastus lateralis at three time points to assess individual cellular and molecular adaptations to resistance training and also changes in response to metformin.

**Discussion:**

The response of aged muscles to a resistance training program does not always result in a positive outcome; some individuals even experience a loss in muscle mass following resistance training. Thus, adjuvant therapies, including pharmacological ones, are required to optimize response to training in those who do not respond and may be at increased risk of frailty. This is the first known metformin repurposing trial in non-diseased individuals, aimed specifically at the resistance exercise “non-responder” phenotype present in the aging population. The overall goal of this trial is to determine if combined exercise-metformin intervention therapy will benefit older individuals by promoting muscle hypertrophy and strength gains, thereby maintaining functional independence.

**Trial registration:**

ClinicalTrials.gov, NCT02308228. Registered on 25 November 2014.

**Electronic supplementary material:**

The online version of this article (doi:10.1186/s13063-017-1932-5) contains supplementary material, which is available to authorized users.

## Background

**Fig. 1:**
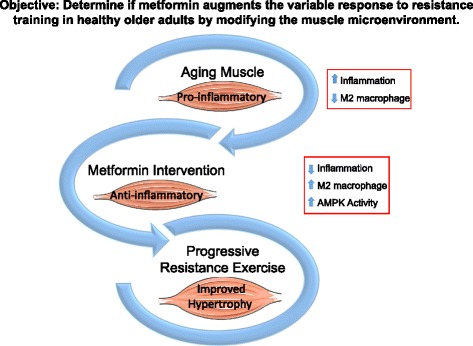
Does metformin augment the variable response to resistance training in healthy older adults by modifying the muscle microenvironment? Study objective for a randomized control trial of metformin to improve the response of muscle to resistance exercise

Muscle mass and strength are critical determinants not only of a person’s quality of life and functional independence, but also metabolic health, as skeletal muscle is the primary regulator of glucose uptake, usage, and storage. The aged suffer obligatory losses of muscle mass and strength, exacerbated by illness and physical inactivity, and in the absence of effective countermeasures, advancing age leads to physical frailty and dependent living. The significance of this cannot be overstated, as the consequent impact on individual life quality and system-wide healthcare expenditures is staggering, and compounded by the ever-expanding elderly population. Muscle mass and strength decrease approximately 10% per decade after the age of 50, with strength loss being even more pronounced after the age of 70 [1]. While resistance exercise training is an established method to increase muscle mass, strength, and functional capacities such as balance and mobility, this effect can be very low in aging cohorts [2, 3]. In fact, up to 38% of older adults do not respond with significant muscle growth when performing resistance exercises alone [4]. Thus, identifying strategies to improve muscle health and applying therapies that may be combined with resistance exercise to slow the debilitating conditions associated with the aging process are of paramount importance. This protocol was designed to study the molecular and cellular mechanisms underlying the “non-responder” phenotype with the goal of identifying a novel intervention to enhance muscle adaptation to progressive resistance exercise training (PRT) in healthy, elderly individuals. A randomized controlled trial is currently underway to study whether metformin, a first-line drug of treatment for type 2 diabetes, can be used alternatively as a treatment to improve the ability of older individuals to respond appropriately to PRT (Fig. 1).

Recent work has shown that advanced age promotes an inflammatory muscle microenvironment which may contribute to the exercise “non-responder” phenotype [5–8]. Pro-inflammatory cytokines such as interleukin-6 (IL-6) and tumor necrosis factor alpha (TNF-α) have elevated expression levels both pre- and post-resistance training in seniors compared to their younger counterparts, with TNF-α being a key prognosticator for non-responders following a 16-week resistance training intervention [6, 9]. Higher levels of these inflammatory cytokines have been associated with lower muscle mass and strength [9]. Additionally, it has been suggested that aging results in substandard muscle macrophage function, hindering the regulation of inflammation and ultimately the regenerative capacity of skeletal muscle [10].

Muscle macrophages have been most studied within the context of muscle damage and regeneration in rodents, where they have been shown to progress from a phagocytic, inflammatory M1 state to an anti-inflammatory M2 state that promotes repair [11–14]. M2 macrophages have also been reported to protect against muscle atrophy in rodents and promote muscle recovery both in vivo and in vitro [15]. Many of the factors produced by M2 macrophages facilitate muscle growth and repair by stimulating the activity of muscle stem cells called satellite cells [16, 17]. The resolution of inflammation following injury in muscle is a product of both repressed inflammatory cytokine production and transcriptional up-regulation of anti-inflammatory genes like insulin-like growth factor (IGF)-1 and IL-4 and IL-10 from M2 macrophages. We showed that resistance exercise in humans that promotes muscle growth results in an increase in the relative proportion of alternatively activated M2 macrophages, with only a small subpopulation expressing classical, pro-inflammatory M1 characteristics 3 days after the exercise bout, and this response was impaired in the elderly [10]. Thus, evidence supports the idea that augmenting M2 macrophage abundance in muscle will facilitate a hypertrophic response.

The use of pharmacological treatments that moderate the inflammatory microenvironment of aged muscle is of particular importance to the success of exercise interventions such as PRT. Metformin has come to the forefront due to recent negotiations undertaken by experts in the aging field with the Food and Drug Administration (FDA) for the classification of an anti-aging drug, whose widespread prescription could benefit our growing aged population [18]. Metformin is a biguanide compound, prescribed since the early 1980s as an anti-hyperglycemic agent for prediabetes and type 2 diabetes. Re-evaluation and potential re-classification stem from longitudinal studies carried out in diabetic patients subject to 10+ years of metformin treatment [19]. The diabetic subjects receiving metformin monotherapy demonstrated a 15% higher survival rate than the aged matched population over the 10+ years under observation [19]. This finding supports the position that metformin treatment may be applicable to improving the health span of non-diabetic populations. Metformin primarily exerts its major effects on improving insulin sensitivity through activation of adenosine monophosphate-activated protein kinase (AMPK), a master switch to regulate suppression of hepatic glucose output [20–22]. Recent work shows that skeletal muscle repair may be improved when AMPK is activated [23], and that the AMPKα1 isoform is required for macrophages to acquire the functions of the M2 subtype in muscle [24]. The AMPKα1 isoform in macrophages is the only catalytic subunit expressed, and when in association with an AMPK activator such as metformin (by way of activating transcription factor 3, ATF-3) or 5-amino-1-β-d-ribofuranosyl-imidazole-4-carboxamide (AICAR), this isoform demonstrates the ability to induce M1 to M2 macrophage polarization such as in response to lipopolysaccharide (LPS), an endotoxin with pro-inflammatory effects, over placebo [25]. When AMPKα1 is absent, skeletal muscle repair is delayed, associated with elevated levels of M1 macrophage markers [24].

**Fig. 2:**
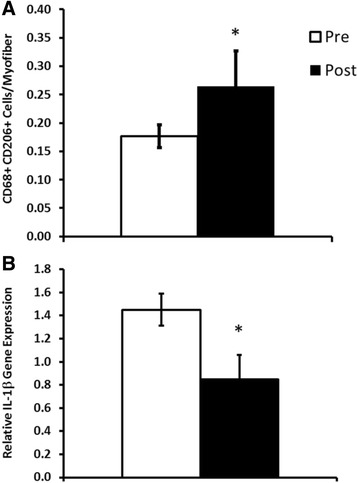
Preliminary analyses of the effects of metformin on muscle tissue. Effects of metformin on muscle tissue M2 macrophage (CD68 + CD206+) frequency (**a**) and IL-1β inflammatory gene expression (**b**). *Different from pre-treatment, *p* < 0.05. Values are mean ± standard error (*SE*)

Interestingly, our preliminary analyses of muscle from insulin resistant participants (*n* = 6) showed that 10 weeks of metformin (1700 mg per day) effectively increased M2 macrophage abundance (Fig. 2a) and decreased inflammatory cytokine gene expression (Fig. 2b) in vastus lateralis muscle biopsies. These provocative findings have led us to our central hypothesis that adjuvant metformin treatment may improve the responses to PRT in the elderly by altering the muscle tissue inflammatory environment, thereby enhancing mechanisms that drive resistance training-induced myofiber hypertrophy.

## Methods/design

### Human subject participation

#### Trial summary and overall design

**Fig. 3:**
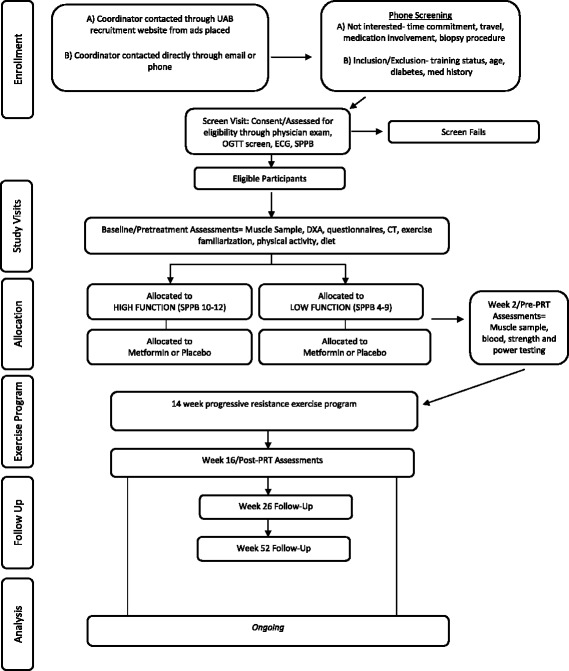
MASTERS Flow Diagram

Subjects are participating at two sites, the University of Kentucky (UK) and the University of Alabama at Birmingham (UAB), in a randomized, double-blind, placebo-controlled trial centered on a novel, alternative use of metformin to potentially augment muscle mass gains to resistance exercise. The two-site design ensures that the recruitment goals are met and that the results are generalizable due to the diverse population that could not be studied at either site alone. Complete participation requires that each individual spend approximately 19 weeks attending 8 study assessments and finishing 42 resistance exercise sessions and, thus, approximately 50 total study visits. This time includes a 2-week pre-treatment or baseline period involving a detailed screening and physical exam for inclusion/exclusion clearance as well as baseline testing for glucose tolerance, body composition, and muscle function, a 2-week medication ramp-up period prior to exercise along with medicationonly testing, followed by 14 weeks of supervised PRT. All post-intervention assessments are completed within 3 days following training. In addition, participants can opt to complete two follow-up assessments of muscle function, quality of life, and physical activity levels at approximately 10 and 36 weeks post-training. The effects of metformin or placebo on the muscle environment after 2 weeks of ramped medication and when combined with 14 weeks of PRT are measured. The protocol is designed in accordance with the Standard Protocol Items: Recommendations for Interventional Trials (SPIRIT) guidelines for interventional trials (Additional file 1). A summary of study visits and the participant data collection schedule are shown in Table 1, and the flow diagram for the overall study in Fig. 3. The study is currently being conducted at each respective Center for Clinical and Translational Science (CCTS) Clinical Research Unit, the Human Performance Lab at UK, and the Center for Exercise Medicine at UAB where subjects are compensated $300 for their time. Subjects are stratified and randomized based on study site and functional status.

**Table 1:** Summary of human subject participation and data collection schedule

Time point	Physical exam and medical history	Blood draw	Oral glucose tolerance test	Physical function testing (SPPB)	Body composition (DXA, CT, and circumferences)	Muscle biopsy	SF-36, PASE, PROMIS modules	Muscle strength and power testing (Biodex, 1RM)	4-day diet record	Physical activity monitoring
Pre-treatment	X	X	X	X	X	X	X	Familiarization	X	X
2 weeks of metformin or placebo treatment only (prior to progressive resistance training, PRT)										
Week 2 (pre-PRT)		X				X		X		
Week 4								X		
Week 9 (mid-PRT)		X (CMP only)						X		
Week 16 (post-PRT)		X	X	X	X	X	X	X	X	X
Long-term follow-up (week 26 and week 52)				X			X	X		X

Each individual can participate in the study for approximately 1 year. Participation includes pre-treatment, medication- or placebo-only ramping period, 14 weeks of PRT, and optional post-training follow-up at 10 weeks (week 26) and 36 weeks (week 52) post-training. Once subjects start medication or placebo treatment, they continue taking the treatment until post-training outcomes are assessed. The scheduling timeline is approximate and allows for the scheduling needs of the subjects and study staff.

*CMP* comprehensive metabolic panel, *SPPB* Short Physical Performance Battery, *DXA* dual-energy X-ray absorptiometry, *CT* computed tomography, *SF-36* Short-Form 36, *PASE* Physical Activity Survey for the Elderly, *PROMIS* Patient-Reported Outcomes Measurement Information System, *1RM* one repetition maximum

#### Recruitment and enrollment

One hundred and twenty men and women ≥65 years of age (*n* = 72 at UAB, *n* = 48 at UK), representative of the racial and ethnic makeup of greater Birmingham, Alabama and greater Lexington, Kentucky, are being recruited through general advertisements and volunteer databases. Given the anticipated attrition rate of 20%, this will allow us to achieve a final sample size of 100. Ethnic and racial groups are expected to enroll based on the proportions of the surrounding areas with emphasis placed on targeted recruitment of under-represented minorities. However, representation of these groups may be lower than intended. Individuals will be recruited who are not currently resistance training or participating in other forms of organized exercise more than two times per week, and who have a Short Physical Performance Battery (SPPB) score >3 (score range 0–12). These represent non-disabled and mobile individuals who are able to participate in the functional testing and resistance exercise. Recruits are pre-screened by telephone interviews to minimize screen failures due to medical history, and all procedures included in the study are explained in detail (including approximate time commitment and potential risks) to the subjects by a member of the research team designated to do so. Upon an initial visit, subjects undergo a detailed medical history, medication use history, resting electrocardiogram, and physical exam by the study physician or physician assistant. Final enrollment decisions and eligibility are based on inclusion/exclusion criteria, 2-h oral glucose tolerance test (OGTT), and bloodwork also performed during the screening visit. These criteria are shown in Table 2.

**Table 2:** Study inclusion and exclusion criteria

Inclusion criteria
• ≥65 years of age
• Independently mobile with an SPPB score 4–12
• Access to transportation
• Capable of providing informed consent (cognitively intact)
• Negative diagnostic (12-lead ECG), submaximal Graded Exercise Testing (GXT) (if applicable)
Exclusion criteria
• Obesity (body mass index (BMI) >30)
• Serum creatinine >1.4 because of risk of lactic acidosis with metformin
• History of structured, regular resistance exercise training within the past year (more than two times per week consistently)
• Chronic aspirin or non-steroidal anti-inflammatory drug (NSAID) use (unless it can be safely stopped prior to the biopsies) and any other use of an anticoagulant (e.g., Coumadin) or history of bleeding
• History of alcoholism or liver disease
• History of hypo- or hypercoagulation disorders including subjects taking Coumadin
• Any end-stage disease and/or a life expectancy less than 1 year
• Neurological, musculoskeletal, or other disorder that would preclude them from completing resistance training and all performance tests
• Uncontrolled hypertension, unstable or exercise-induced angina pectoris or myocardial ischemia, or congestive heart failure
• Diabetes mellitus: HgbA1C >6.5, or fasting glucose >126 mg/dl, or 2-h glucose (on oral glucose tolerance test (OGTT)) >200)
• Any other medical condition that would interfere with testing or increase one's risk of complications during exercise, as judged by the study physicians
• Any other condition or events considered exclusionary by the Principal Investigator and/or physician, such as non-compliance
• Lidocaine allergy (1% lidocaine is the local anesthetic used during the muscle biopsy procedure)
• Currently receiving androgen or anabolic therapy

#### Physical function

Participants complete the SPPB for the assessment of physical function [26]. This battery of tests includes three timed standing balance tests (side-by-side, semi-tandem, and tandem), a 3-or 4-m habitual gait speed test performed twice, and a timed repeated chair sit to stand (five times). Performance for each set of tasks is scored (0–4), with a summary score of 0–12. Functional assessment data are collected during pre-treatment, post-training, and during optional follow-up testing and are used as a stratification measure at each site (high vs. low).

#### Randomization and stratification

Subjects screening into the study are randomized and stratified by both site (UK vs. UAB) and by SPPB score (4–9 low, vs. 10–12 high) to receive either metformin or placebo. Since recruitment at UAB is expected to be more than at UK, participants will be stratified by site prior to randomization. Randomization is implemented in permuted blocks of 4 using SAS v9.2 PROC PLAN to ensure adequate distribution of all groups across the collection period. Only the statistician and the investigational pharmacy will have access to the randomization and stratification scheme; thus, assignments will be unknown to the investigators and study team. The protocol also appoints an individual outside of study assessments or specimen analysis to reveal group placement to participants at a specified time.

#### Medication or placebo

The metformin and placebo tablets were obtained commercially through different facilities. The placebo tablets were obtained from the Veterans Affairs (VA) Cooperative Studies Program Clinical Research Pharmacy Coordinating Center, which is in New Mexico working through the Biomedical Research Institute of New Mexico. This program is registered with the FDA as a manufacturing, packaging, and distribution facility and serves the needs of VA studies, as well as National Institutes of Health (NIH)-funded and some industry studies. There are many different manufacturers of metformin, and the VA facility in New Mexico has provided us with a tablet which is identical to metformin 850 mg tablets that are manufactured by Amneal Pharmaceuticals, an established generic drug maker. Therefore, we purchased generic Amneal 850 mg metformin tablets through our investigational pharmacy. Subjects randomized to metformin will take increasing doses during the medication ramp-up period of 2 weeks to reduce gastrointestinal (GI) side effects as follows: 1 tablet (850 mg) per day for a period of 7 days and 2 tablets per day (1700 mg) for a period of 7 days, the latter being the target clinical dose, continued throughout the 14 weeks of PRT and final week of post-intervention assessments. In some subjects, the dose progression may be slower and may not reach 1700 mg/day due to GI side effects. The study physician makes individualized modifications should the need arise. Subjects remain in the study as long as they can tolerate at least 850 mg/day. These data will be collected and reported. The placebo tablets look identical to the metformin tablets, but contain inert substances, and the escalating dose schedule is the same. The hospital investigational pharmacy dispenses the metformin or placebo in uniform generic bottles at five time points (pre-treatment for initial 2-week medication ramp, baseline PRT weeks 2–4, PRT weeks 4–8, PRT weeks 8–12, and PRT weeks 12–16) throughout the protocol so that compliance can be monitored. This allows for a compliance schedule of approximately 4 weeks where subjects return any unused product. Extra doses are given as needed and are noted by the study coordinator.

#### Quality of life

Quality of life is being determined through self-report instruments, the Short Form 36 (SF-36) and the Patient-Reported Outcomes Measurement Information System (PROMIS), for the effectiveness of the exercise training intervention on different health-related domains including physical, mental, and social well-being. Subjects complete these assessments during pre-treatment, following the resistance exercise program, and during follow-up assessments at 10 and 36 weeks post-training.

#### Physical activity measures

The Physical Activity Survey for the Elderly (PASE) as well as physical activity monitors are used in this study. The PASE is a self-report or interview-based measure designed to capture and assess occupational, household, and leisure activities typically performed by older adults including those of lighter intensity. Time spent participating in each activity area is multiplied by a weighted value that reflects the amount of energy expended by an older person engaged in that activity. These weighted values are then summed to yield a composite PASE score which is used for data analysis. This questionnaire has been found to be both reliable and valid among community-dwelling and physically disabled older adults. The physical activity monitor being used in this study is the Fitbit Flex; it is worn on the non-dominant wrist for an assessment of daily step counts over a period of at least 5 days. The day given and day of return are excluded from the analysis, since the subject will not have worn the monitor for a complete day. The measures are taken before resistance exercise training and during the last week of training to account for possible increases in activity due to the exercise sessions. Subjects also complete these measures during their optional follow-up visits by wearing the monitors for a period of 1 week before returning to complete strength and functional measures.

#### Dietary monitoring

Participants are asked to maintain their normal dietary intake throughout the study period. Energy intake and macronutrient composition are assessed by 4-day diet records during pre-treatment and during the last week of training. The nutrient content is determined by those qualified to use the Nutrition Data System for Research, which utilizes the multiple-pass method to help improve the validity of dietary data [27].

#### Progressive resistance exercise training (PRT)

The 14 weeks of PRT (42 sessions ±5 sessions), summarized in Table 3, are supervised by trained personnel such as exercise physiologists or senior-level physical therapy or athletic training graduate students under the supervision of an exercise physiologist. Each site has a certified, lead exercise physiologist with several years of experience, responsible for managing the day-to-day activities and supervising all trainers. Subjects are instructed on proper techniques and continuously monitored. Following a 5-min warm-up on a self-selected bicycle ergometer or treadmill, PRT consists of eight constant load movements to train all major muscle groups. These exercises include those to strengthen the lower body and thigh muscles (leg press, knee extension, body weight squat progressing to a split squat if the participant is able, calf press) as well as upper body exercise (chest press, lateral pulldown, biceps curl, and triceps pressdown). Core and trunk exercises are also a part of the routine, including abdominal work (various abdominal exercises) and lower back flexibility and strengthening (alternating supermans). All resistance exercises are performed bilaterally, progressing to reach full volume and intensity for each exercise by the end of the second week. We are implementing a variable-intensity prescription: “high-low-high” 3 days/week program which has previously been shown to optimize muscle mass and strength gains in older adults [28]. On Mondays and Fridays, intensity is high with subjects completing 3 sets of 8–12 repetitions at 10RM with 60–90 s between sets. Progression is incorporated continuously by incrementing the resistance load when 12 repetitions are completed for 2 of 3 sets. On Wednesdays, resistance loads are reduced ~30% with 30- to –60-s rest periods with the emphasis on more rapid, concentric training (with controlled eccentric loading) to develop explosive power while providing a protracted recovery period between high resistance sessions. Participants complete the routine in supersets combining two exercises, loading antagonistic or uninvolved muscle groups, in succession with minimal rest between exercises and 60- to 90-s rest periods between supersets. Following resistance exercise, participants complete a 5-min cooldown. Participants aim to complete 42 exercise sessions but have the flexibility to train ±5 sessions to account for follow-up testing as well as for participant schedules and vacations, etc. Participants are expected to be in good compliance by completing at least 2 consecutive exercise sessions in a row before follow-up testing. Note that, due to the two-site design, exercise equipment between the sites is different, with UK utilizing pneumatic air-driven Keiser equipment and UAB utilizing standard plate-loaded and weight stack BodyMasters and LifeFitness equipment. Between-site analysis of training outcomes will be performed to account for training effects due to differences in equipment. Details of the resistance training protocol are given in Table 3.

**Table 3:** Progressive resistance training protocol

14 weeks (42 ± 5 sessions)			
	Monday	Wednesday	Friday
Goal	Hypertrophy	Power	Hypertrophy
Intensity	∼70% 1RM	∼40% 1RM	∼70% 1RM
Reps	8–12	12	8–12
Sets	3	3	3
Rest	∼60 s	∼30 s	∼60 s

Each session includes eight exercises performed bilaterally in pairs, with indicated rest between pairs (chest press/squat, leg press/calf press, lateral pulldown/leg extension, bicep curl/tricep extension), along with core and trunk exercises. Progression to 3 full sets is achieved by session 5. Initial exercise intensity is determined by 1RM for the chest press, leg press, and leg extension, while a 10RM is used for all other exercises. *RM* repetition maximum

#### Muscle biopsy

Subjects have three biopsies during this protocol: at pretreatment, after 2 weeks of metformin or placebo, and at3 days following the last resistance exercise bout. Subjects taking blood thinning medications are asked to stop those medications for a period of 3–5 days prior to each biopsy. Muscle tissue is obtained from the vastus lateralis after administration of local anesthetic (1% lidocaine premixed with bicarbonate) using a 5 mm Bergstrom needle with suction. A small incision is made in the skin to allow the needle to be briefly inserted into the muscle so as to obtain approximately 200–300 mg of tissue, usually occurring over two passes. Direct pressure is applied to stop bleeding for approximately 5–7 min, and the wound is closed with Steri-Strips and covered with gauze and a pressure bandage. Biopsies follow a left, right, left leg pattern unless a research subject requests differently. Muscle tissue is divided as follows: 100 mg is processed for muscle cell isolation, ~100 mg is snap-frozen (~30 mg aliquots) in liquid nitrogen for RNA and protein isolation, and ~50 mg is mounted in tragacanth gum and frozen in liquid nitrogen-cooled isopentane for detailed immunohistochemical (IHC) analyses. All biopsies are performed with the subjects fasting.

#### Post-training follow-ups

The subjects remain in the study after completion of the training program for an additional 36 weeks to be contacted for optional follow-up visits. These visits occur at approximately 10 weeks and 36 weeks following the training period but are optional for the subjects. The retention of strength, function, physical activity, and quality of life is examined. Subjects are not asked to refrain from any activity they would like to participate in including continuing their own strength training. Metformin or placebo is discontinued during this time. Changes in muscle strength and power are plotted against all time points shown in Table 1. We assess strength change from the leg extension exercise due to its isolation of the vastus lateralis and the leg press as a secondary measure. Muscle power is assessed from the isotonic mode on the Biodex set at 40% of each subject’s maximum voluntary isometric contraction. Insulin sensitivity is measured from plasma glucose and insulin values during a 2-h OGTT. The Homeostatic Model Assessment of Insulin Resistance (HOMA-IR) is calculated from fasting blood samples while the Matsuda index is utilized for all other samples.

#### Data safety monitoring

The current clinical trial has obtained the required institutional review board (IRB) approval. Both sites have conducted similar trials in the past and are experienced with all regulatory requirements and with working with their IRBs. The study has eligibility requirements to ensure that subjects enrolled have an appropriate risk-to-benefit ratio. The Principal Investigators (PIs) are ultimately responsible for keeping all study documents updated and available for inspection by the sponsor, the UAB and UK IRBs, and other authorized reviewers. Both sites report in the same manner and in the same time frame. Monitoring for adverse events (AEs) is conducted in real time by the study investigators and study coordinators. Risks involved with this study are considered greater than minimal risk and are listed in the consent form. For this reason, we have utilized the standing independent Data Safety Monitoring Board (DSMB) as chartered by the UK CCTS to monitor the safety of this study at both sites. The DSMB reviews protocol performance, regulatory requirements, particularly the reporting of AEs, and serves as the sole DSMB for the study. Both sites use the same standardized AE report for the DSMB review to allow for an effective assessment of potential issues. The DSMB review is given to the PIs and study coordinators, who can then report to the UK and UAB IRBs during Continuation Reviews.

The study coordinator is in constant contact with subjects to assess pain, infection, and other symptoms indicating possible post-procedure AEs. Subjects are discharged from the Clinical Research Unit with specific self-monitoring guidelines and instructed to call immediately regarding any concerning signs or symptoms. The study procedures are halted at any time a serious safety concern is noted.

AEs are graded according to intensity and relatability to the study. Annual reporting of AEs and serious adverse events are conducted with the IRB Continuation Review in their appropriate time frames according to their protocol.

### Outcome measures and analyses

#### Primary outcomes

The primary outcomes are as follows:

1. Muscle size
  - Myofiber cross-sectional area (CSA)
  - Computed tomography (CT) skeletal muscle area of the right thigh
  - CT skeletal muscle area of the vastus lateralis
2. Muscle strength and power
  - 1 RM on the leg extension exercise
  - Biodex maximum voluntary isometric contraction (MVIC)
  - Biodex isotonic power at 40% MVIC

##### Muscle size

This involves individual fiber CSA and total thigh muscle size/mass. The CSA at the myofiber level from vastus lateralis muscle biopsies is the primary outcome of interest. Fiber type-specific CSA is quantified on 7-μm serial cryosections using an antibody- recognizing laminin to delineate individual myofibers, followed by incubation with a battery of monoclonal antibodies against the different myosin heavy chain (MyHC) isoforms. The three MyHC primary antibodies (types I, IIa, and IIx) are of different isotypes so that all primary antibodies are added to the sections simultaneously followed by isotype-specific secondary antibodies conjugated to different fluorescent tags. Digital images are captured of the entire cross section (between 400–1200 fibers), and mean myofiber CSA by fiber type quantified using a recently developed automated algorithm.

Muscle size is determined using a single slice CT image collected on a GE Discovery CT750 HD at UAB and Siemens Somatom Definition at UK at the mid-thigh defined as the midpoint between the inguinal crease and the proximal border of the patella with the hip and knee flexed ~90°. CT images are used to quantify skeletal muscle and fat area of the right thigh of each subject using 100 mA with a scanning time of 3 s and a 512 × 512 matrix. With the subject supine, one 5-mm-thick cross section scan of the leg is taken by lining the scan to the midpoint mark identified on each participant. The feet of each participant are wrapped to minimize movement. Tissue area quantification is determined using corresponding attenuation values of ≥200HU; –190 to –30 HU; and 0–100 HU for bone, adipose tissue, and skeletal muscle, respectively, using available software (NIH ImageJ; http://rsbweb.nih.gov/ij/). Skeletal muscle is subdivided into areas of low attenuation (0–34 HU) representing fat-rich muscle, and high attenuation values (35–100 HU) representing muscle with normal fat content. Each subject receives a CT scan during pre-treatment and 1–3 days following the last resistance training session. The total skeletal muscle areas of the right mid-thigh, quadriceps muscle area, and isolated vastus lateralis are quantified.

##### Muscle strength and power

Voluntary, dynamic strength is evaluated by testing a one repetition max (1RM), defined as the maximal load that a subject can lift one time with proper form through a full range of motion, via our well-established methods that have been standardized across sites [8, 29, 30]. After 2 sets of warm-ups at an estimated 40–50% for the first set and 70–80% for the second set, single repetition trials, separated by 1–2 min of rest, are performed with increasing resistance until two failed attempts at a given load. The last successfully lifted load with good form and range of motion will be recorded as the 1RM. Verbal encouragement is given during all lifts, and 1RM is always completed during high or heavy days as described above.

We also evaluate MVIC knee extension strength with a knee angle of 60° using our established methods on a Biodex 4 dynamometer, available at both sites [31]. One set of 5 reps is also being completed by the subjects to determine knee extension concentric power using the Biodex set on isotonic mode to allow for variable velocities with a constant external load equal to 40% of maximum voluntary isometric strength. Peak power is recorded as the highest power recorded during the 5 repetitions. Subjects complete these assessments 5 times throughout the protocol including a familiarization session during pre-treatment, after 2 weeks of metformin or placebo, after 2 weeks of resistance training (week 4), at the midpoint of the resistance training program (week 9), and during their last week of training (week 16). Familiarization sessions are designed to accustom the subjects to the different exercises using proper technique and explain 1RM testing (maximum efforts were not given on 1RMs). However, maximum efforts are given on the Biodex to be used for comparison with the effort given during the 2-week metformin or placebo testing period. Week 4, after 2 weeks of resistance training, is used as the baseline 1RM strength to account for the initial neuromuscular adaptations occurring at the beginning of the resistance training program.

#### Secondary outcomes

Secondary outcomes include the following:

1. Body composition: dual-energy X-ray absorptiometry (DXA) total and thigh mineral-free lean mass, waist, abdominal, and hip circumferences
2. Insulin sensitivity
  - Fasting plasma glucose and insulin (HOMA)
  - Matsuda index based on glucose and insulin values
3. Physical activity and quality-of-life self-reports

##### Thigh muscle mass

Regional thigh DXA mineral-free lean mass is used as a secondary indicator of muscle response. DXA scans are performed for body composition (whole body, regional fat, and lean mass) and bone mineral density assessments using a Lunar Prodigy (UAB) and an IDXA (UK) using standardized methods for regional partitioning. Data quality is assured by phantom calibrations, and each participant receives a DXA scan at two time points during the study, pre-treatment and post-resistance training, by study personnel trained in this procedure. The subjects are instructed to remove all objects such as jewelry or eyeglasses and to wear a hospital gown, or a lightweight shirt and shorts containing no metal during the scanning procedure. All scans are analyzed by a trained and certified investigator using the GE Lunar software v10.0. DXA bone mineral content (BMC; kg), DXA bone mineral density (BMD; g/cm^2^), DXA fat-free mass (FFM; kg), DXA mineral-free lean mass (MFL; kg), DXA fat mass (Fat; kg), and DXA percent fat (%Fat) are assessed. Furthermore, custom analyses are performed to determine femur length and right and left thigh muscle and fat mass. Femur length is measured from the center of the junction of the femoral head (at the femoral neck) and the acetabulum to the center of the bottom of the medial condyle. Right and left thigh muscle and fat mass are calculated by subtracting the lower leg from the respective right or left leg total mass by creating a custom region of interest (ROI) through the center of the knee joint between the tibial plateau and the femoral condyles and encapsulating the lower leg past the toes. In addition to DXA, circumferences are taken at three sites using American College of Sports Medicine (ACSM) standardized procedures including the waist defined as the narrowest part of the torso, the abdomen at the level of the umbilicus, and the hips defined as the maximal circumference of the buttocks.

##### Fasting blood glucose and insulin and oral glucose tolerance test (OGTT)

Subjects undergo four blood draws and complete two OGTTs in order to assess fasting glucose and insulin, and also to assess safety and eligibility requirements (i.e., creatinine, glucose, liver enzymes, lipids, thyroid-stimulating hormone (TSH), complete blood count (CBC) with platelets), as well as banking. A standard 2-h OGTT is performed after an 8- to 12-h fast using 75 g of glucose. Blood is drawn before and at 30, 60, 90, and 120 min after ingestion of the glucose load. The Matsuda index, which correlates well with the euglycemic clamp, is used to calculate insulin sensitivity [32]. Subjects are required to be fasting during each of these research visits.

#### Exploratory outcomes

The following exploratory outcomes are included:

1. Macrophage profiling
  - Macrophage abundance
  - Macrophage polarization state
2. Satellite cells
  - Abundance
  - Activation state
  - Fusion
3. Assessment of inflammation
  - Cytokine gene expression
  - Signal transduction

##### Macrophage profiling

Resident muscle macrophage number and polarization state at baseline (first biopsy), after metformin or placebo only (second biopsy), and after 14 weeks of PRT (third biopsy) are determined with IHC. Subjects are asked to continue their normal activities of daily living but to refrain from any unaccustomed activity or exercise, including resistance training, through the first 2 weeks of the protocol to prevent any exercise-induced muscle inflammation. Pan monocyte/macrophage antibody co-stains of CD11b and CD68, together with 4′,6-diamidino-2-phenylindole (DAPI) staining, are used to quantify total macrophages in 7-μm cryosections. The relative frequency of CD11b+/CD206-/CD163- M1 pro-inflammatory macrophages to CD11b+/CD206+ M2 alternatively activated macrophages is also quantified. CD163 is used for the confirmation of CD206, M2 macrophages. The relative frequency of macrophage subtypes is then expressed per fiber area/total fibers.

##### Satellite cell analyses

Muscle stem cell (satellite cells) abundance per fiber is quantified by IHC with the Pax7 monoclonal antibody. MyoD is expressed specifically in activated satellite cells, and IHC analysis of MyoD is used to identify activated satellite cells. These analyses are combined with counting total myofiber nuclei (DAPI-stained nuclei residing within the dystrophin-labeled sarcolemma) to monitor myonuclear accretion from satellite cell fusion that accompanies hypertrophic growth of myofibers in humans, including older adults.

##### Assessment of inflammation

We are quantifying inflammatory gene expression in all subjects using the Nanostring nCounter analysis system. Approximately 100 genes are analyzed based on previous work of the genes that were most differentially expressed between the exercise responders and non-responders. Signaling pathways such as the AMPK pathway are measured by western blot using phospho-specific antibodies to Thr172 on AMPK. The mTORC pathway, antagonized by AMPK, is also analyzed, as it plays an important role in regulating protein synthesis in muscle. Other signaling pathways, such as p38 and PKC, are assessed in relation to growth and macrophage profile. Down-regulation of inflammatory signaling proteins, in particular, NFκB and STAT, will also be quantified.

### Statistical plan

We intend to recruit 120 participants to account for a 20% attrition rate (84 UAB and 36 UK) to achieve a final sample size of 100 (50 metformin, 50 placebo). This allows sufficient power for “as observed” comparisons. Endpoints (myofiber CSA, thigh muscle CSA, strength, power, and macrophage abundance) and changes with training will be treated as continuous variables, summarized with descriptive statistics. SAS v9.2 or higher is being used for all analyses, and a significance level of 0.05 is used for all statistical tests. In the case that endpoints are found to be non-normal, appropriate transformations are employed and non-parametric tests used. The primary analysis and representative measure used to calculate power is the comparison of change in type II myofiber CSA for those randomized to either PRT with placebo or metformin. Type II myofiber CSA changes due to PRT with placebo or metformin will also be compared across sex. A two-sample *t* test will have at least 80% power to detect an effect size of 0.6 when the sample size is 50 per group (*n* = 100), assuming a two-sided significance level of 0.05. Based upon our prior resistance training trials in older adults, we expect 14 weeks of PRT alone to be associated with an approximately 20% increase in type II myofiber CSA in both men and women. We predict that adjuvant metformin will yield an additional 25% increase in myofiber CSA (above the 20% due to PRT alone). Prior work (25 men and 25 women, age 65–80 years) showed pre-treatment type II CSA means (SD) of 4095 (1213) μm^2^ in men and 2458 (690) μm^2^ in women. With a 20% increase in PRT alone and an additional 25% increase with metformin, the mean difference between treatment groups would be at least 730 μm^2^, assuming a common SD of 950. The effect size is expected to be at least 0.76, which is larger than the detectable effect size for our planned final sample of 100.

### Statistics specific to primary outcomes

The primary endpoint is the change in type II myofiber size after training, with the secondary outcome being change in muscle size. The primary analysis design is the intention-to-treat (ITT) comparison of change in myofiber/muscle size for those randomized to either placebo or metformin. It is expected that the randomization will lessen the need for covariate-adjusted analyses; however, in the event that adjusted analyses are necessary, a secondary comparison of the change in endpoints for the two groups will be made using analysis of covariance (ANCOVA). Potential confounders include baseline values, BMI, age, race, ethnicity, gender, changes in insulin sensitivity, and pill count. In addition to changes in muscle size, changes in strength are also of interest. Strength is measured at weeks 2, 4, 9, and 16 (training begins after 2 weeks of metformin or placebo). The primary analysis for strength is conducted using the same strategy as for muscle size, using a change score from week 4 (true strength baseline) to 16. However, since additional time points are collected that investigate changes in strength over time between groups, the interaction of group and time can also be analyzed using covariance pattern models (or linear mixed models), where the correlation between observations measured over time can be handled more flexibly (i.e., unstructured and autoregressive variance-covariance matrices).

The ITT analysis is performed using all randomized participants regardless of loss to follow-up, where data for those lost is imputed using last observation carried forward (LOCF). In this case, LOCF should provide a conservative estimate of the effect; however, multiple imputation will also be used to impute missing values and to assess the sensitivity of the results based on LOCF. It is expected that subjects may not be compliant to the prescribed dosing, which is measured by pill count at specified visits. Non-compliant subjects are included in the ITT analysis; however, a modified ITT may also be conducted utilizing an ITT randomization principle but limited to those who achieved at least 1000 mg per day. Thus, analyses are conducted comparing groups without imputed data (“as observed”), which will be impacted by attrition as well as ITT analysis using the randomized sample in which all participants are used, potentially allowing us to detect a smaller effect size (approximately 0.5 if *n* = 120) with 80% power.

## Discussion

As metformin is increasingly being used to treat medical conditions other than type 2 diabetes, identifying novel mechanisms of action of metformin is timely. While the mechanisms of action of metformin are not fully understood, much of the previous research has shown that metformin activates the enzyme AMPK, shown to be influential in glycemic control, energy balance, and metabolism in multiple peripheral tissues [33]. More recent animal research has shown metformin’s profound effects on skeletal muscle through activation of AMPK. Improvements in structural integrity, oxidative metabolism, resiliency to muscle damage, and macrophage polarization have all been reported [34–36]. However, mouse models have shown that metformin can maintain its hypoglycemic effect in the absence of AMPK in the liver, indicating that metformin may act in an AMPK-independent manner [37]. Furthermore, metformin was shown to enhance mitochondrial respiration in skeletal muscle of AMPK-deficient mice after just 2 weeks of treatment [38]. Based on the wealth of research on the beneficial skeletal muscle effects and modulation of a variety of other conditions such as cardiovascular disease, cancer risk, and longevity, metformin has been proposed as a potential anti-aging drug [19, 39–41].

AMPK signaling also responds to exercise in skeletal muscle, but in an age- and sex-dependent manner. It has been demonstrated that AMPK activation is significantly increased in men but not in women following a bout of continuous submaximal aerobic exercise [42] and that aged animals show a reduced AMPK response to exercise [43]. Thus, results from this study will determine if metformin can augment the benefits of exercise in the elderly in an AMPK-dependent or AMPK-independent manner, and if this differs by sex. Results will also provide information on metformin tolerance and the frequency of GI side effects in both male and female healthy elderly individuals.

This will be the first metformin repurposing trial to test the potential synergistic impact of combined exercise-drug therapy on muscle mass and function, and it will be conducted in an aging cohort in need of a treatment that maximizes muscle regrowth and strength gain. This study has the potential to advance our understanding of the mechanisms involved in muscle adaption and to predict those individuals who may have trouble responding to an exercise training program. Age-related muscle inflammation susceptibility is a novel concept [6], which the study proposes as a central mechanism underlying the blunted responsiveness of many older adults to resistance training by promoting a catabolic environment. Our combined intervention will determine if this combined exercise-drug therapy will overcome the variable and non-responsive phenotype seen in aging. By imposing a two-site collaboration, the study becomes more generalizable and contains sufficient power not normally found in smaller resistance training trials.

In conclusion, there are several innovative features of the proposed experiments that are expected to significantly advance the field and improve muscle regrowth and mobility, with the overall goal of reducing risk of disability among older adults. The proposed work is expected to have a powerful impact, as we will be the first to determine whether metformin, in combination with resistance exercise designed to elicit muscle hypertrophy, will augment progressive resistance training-induced muscle gains in older adults and successfully restore function, health, and quality of life.

### Trial status

The study has been active and open for enrollment since November 2014 with an anticipated completion date of December 2017.

## Additional file

Additional file 1: SPIRIT checklist. (DOC 121 kb)

## Abbreviations

ACSM: American College of Sports Medicine
AE: Adverse event
AMPK: 5′ adenosine monophosphate-activated protein kinase
ATF-3: activating transcription factor 3
BMC: Bone mineral content
BMD: Bone mineral density
CBC: Complete blood cell count
CCTS: Center for Clinical and Translational Science
CSA: Cross-sectional area
CT: Computed tomography
DAPI: 4′,6-diamidino-2-phenylindole
DSMB: Data Safety and Monitoring Board
DXA: Dual energy x-ray absorptiometry
FFM: Free fat mass
FM: Fat mass
HIPAA: Health Insurance Portability and Accountability Act
HOMA: Homeostatic Model Assessment
HU: Hounsfield unit
IGF-1: Insulin growth factor 1
IHC: Immunohistochemistry
IL-10: Interleukin-10
IL-1β: Interleukin-1beta
IL-4: Interleukin-4
IL-6: Interleukin-6
IRB: Institutional review board
ITT: Intent to treat
LOCF: Last observation carried forward
LPS: Lipopolysaccharide
MFL: Mineral-free lean
mTORC: Mechanistic (formerly mammalian) target of rapamycin complex
MVIC: Maximum voluntary isometric contraction
MyHC: Myosin heavy chain
OGTT: Oral glucose tolerance test
PASE: Physical Activity Survey for the Elderly
PROMIS: Patient-Reported Outcomes Measurement Information System
PRT: Progressive resistance training
RM: Repetition maximum
ROI: Region of interest
SF-36: Short Form 36
SPPB: Short Physical Performance Battery
TNF-α: Tumor necrosis factor alpha
TSH: Thyroid-stimulating hormone
UAB: University of Alabama at Birmingham
UK: University of Kentucky
VA: Veterans Affairs
